# Sustainable and Simple Water‐Induced Separation of Ionic Liquid Mixtures

**DOI:** 10.1002/anie.202503863

**Published:** 2025-05-08

**Authors:** Beatriz R. de Moraes, Agilio Pádua, Rômulo A. Ando, Margarida Costa Gomes

**Affiliations:** ^1^ Laboratoire de Chimie ENS de Lyon and CNRS 46 allée d'Italie Lyon 69364 France; ^2^ Laboratório de Espectroscopia Molecular Instituto de Química Universidade de São Paulo Av. Prof. Lineu Prestes, 748 São Paulo 05508‐000 Brazil

**Keywords:** Ionic liquids, Phase diagram, Recycling, Thermodynamics, Water‐induced phase separation

## Abstract

Ionic liquids (ILs) are often considered inseparable once mixed, posing a major challenge for their reuse and recycling. We demonstrate a simple and effective method to separate ILs containing hydrophobic ([NTf_2_]^−^) and hydrophilic ([OAc]^−^) anions under ambient conditions. This process is driven by the thermodynamic behavior of the ILs in presence of water that enables the near‐complete (⩾99%) recovery of pure components without complex purification steps. Our findings prove that pure ILs can easily be recovered after being mixed and open new pathways for the sustainable use of ionic fluids in various applications.

## Graphical abstract

The cover image illustrates an innovative method for recovering ionic liquids (ILs) from their mixtures. This approach enables the recovery of over 99% of the IL by simply adding water–abundant, and safe solvent–under mild conditions, all while preserving the integrity of the ILs. This method marks the beginning of a new field in IL recycling, driven by the strategic balance of hydrophilicity among the components of the IL mixture.

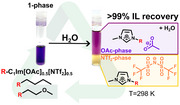

Ionic liquids (ILs) are versatile for applications as solvents, electrolytes, or as reaction media because they can be designed by the judicious choice of their constituent ions and functional groups. Mixtures of ILs^[^ ^1^ ^]^ offer additional possibilities for fine‐tuning physical and chemical properties by varying not only the selection of cations and anions but also their relative proportions.

Mixing ILs can create complex ionic networks that are predominantly governed by Coulomb interactions that determine their liquid‐phase properties.^[^ ^2^, ^3^ ^]^ Although mixing ILs offers a straightforward strategy to achieve specific functionalities, separating ILs mixtures poses significant challenges due to their inherently nonvolatile nature. So far, proposed solutions to recover the pure ILs after mixing often involve high‐vacuum distillation at elevated temperatures.^[^ ^4^ ^]^ However, operation conditions can lead to the decomposition of certain ILs, particularly those containing carboxylate anions.^[^ ^5^ ^]^ If ILs mixtures are to be used at larger scales, efficient separation of the components and recycling of the ILs are essential towards both economic and environmental sustainability. Promising solutions must reduce energy consumption, avoid harmful solvents, and employ standard chemical equipment in the separation processes.

We demonstrate herein that pure ILs can be recovered with over 99% efficiency from homogeneous salt mixtures through the simple addition of water—a nontoxic, inexpensive, and safe solvent. Our method is illustrated using two homogeneous mixtures, each comprising two ILs with a common cation. The first mixture contains 1‐butyl‐3‐methylimidazolium acetate ([C_4_C_1_Im][OAc]) and 1‐butyl‐3‐methylimidazolium bis(trifluoromethylsulfonyl)imide ([C_4_C_1_Im][NTf_2_]) while the second is composed of 1‐methoxyethyl‐3‐methylimidazolium acetate ([(C_3_O)C_1_Im][OAc]) and 1‐methoxyethyl‐3‐methylimidazolium bis(trifluoromethylsulfonyl)imide ([(C_3_O)C_1_Im][NTf_2_]). The separation process involves adding water to the mixtures, which remain homogeneous until a maximum water concentration is reached, similarly to what was found previously for water‐IL mixtures.^[^ ^6^, ^7^ ^]^ Beyond this point, the system separates into two liquid phases: an upper, lower‐density phase enriched in [OAc]^−^ and a lower, denser phase containing the [NTf_2_]^−^ salt. This phase separation is illustrated in Figure 1 for the mixture [C_4_C_1_Im][OAc]_0.5_[NTf_2_]_0.5_ in the presence of alizarin red S dye.

**Figure 1:**
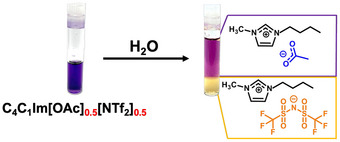
Mixture of [C_4_C_1_Im][OAc]_0.5_[NTf_2_]_0.5_ at 295(1)K and ambient pressure with alizarin red S dye (added to facilitate the visualization) showing pH‐dependent color variation.^[^ ^8^ ^]^ On the left is shown the homogeneous mixture and on the right the biphasic system with an [OAc]^−^‐rich top phase and an [NTf_2_]^−^‐rich bottom phase.

Figure 2 presents the ternary phase diagrams for two IL mixtures with water: [C_4_C_1_Im][OAc] + [C_4_C_1_Im][NTf_2_]  +  H_2_O and [(C_3_O)C_1_Im][OAc]  +  [(C_3_O)C_1_Im][NTf_2_]  +  H_2_O, at 298.0(1) K at atmospheric pressure. The composition of each phase was determined using ^1^H‐NMR spectroscopy and verified through coulometric Karl Fischer titration (see Figure S1 in the ESI). Detailed experimental procedures, including mixture preparation and characterization of homogeneous and biphasic systems, are provided in the ESI.

**Figure 2:**
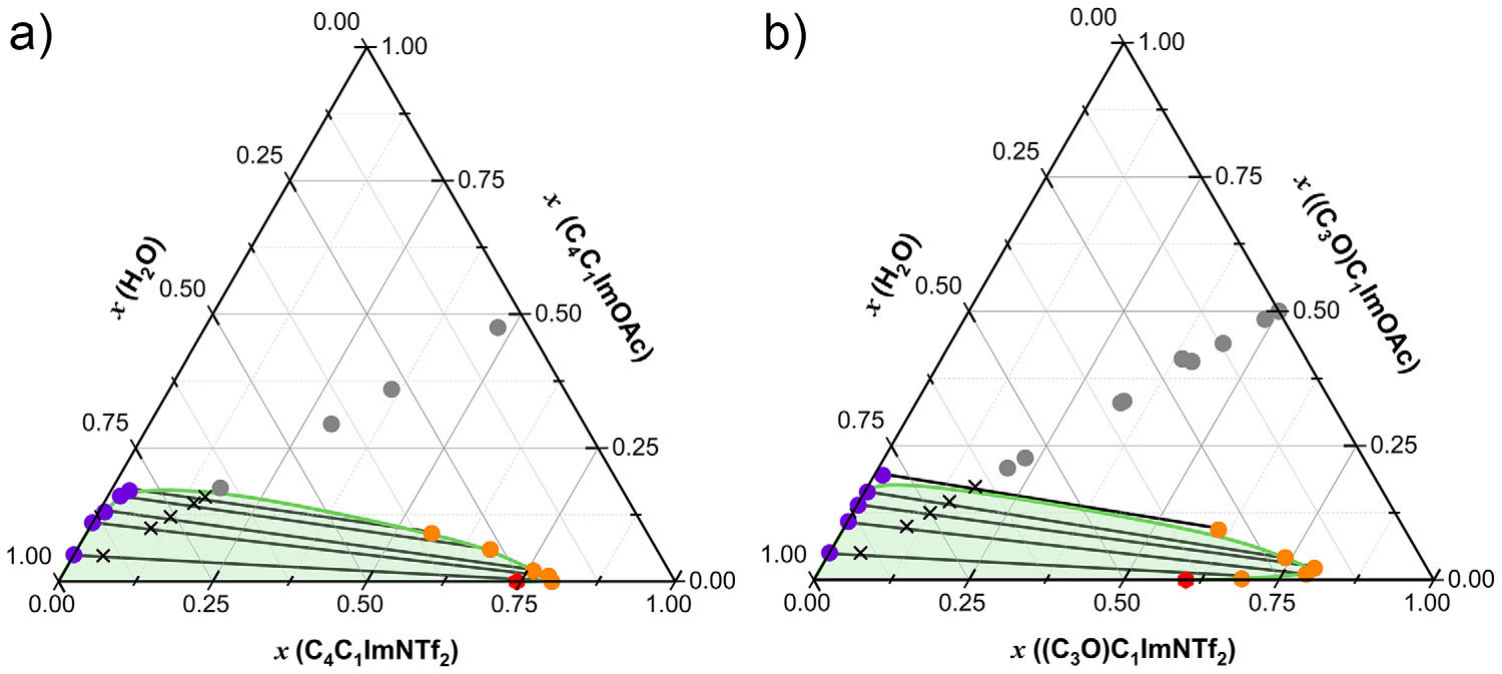
Experimental ternary diagram of (water + acetate‐based IL + NTf_2_‐based IL) at 298.0(1) K for a) [C_4_C_1_Im][OAc]_0.5_[NTf_2_]_0.5_ and b) [(C_3_O)C_1_Im][OAc]_0.5_[NTf_2_]_0.5_ varying composition of the mixture upon the addition of water. The neon green line, marks the boundary between the homogenous and heterogenous phase, was drawn by connecting the experimental points with a B‐spline curve. The red point indicates the solubility limit of water in the pure [NTf_2_]^−^ ILs.

The mixtures are fully miscible with water up to mole fractions *x*(H_2_O) = 0.65 for [C_4_C_1_Im][OAc]_0.5_[NTf_2_]_0.5_ and *x*(H_2_O) = 0.60 for [(C_3_O)C_1_Im][OAc]_0.5_[NTf_2_]_0.5_. As the water content increases, the purity of each phase improves, highlighting the stronger water affinity of the [OAc]^−^ anion compared to [NTf_2_]^−^. At higher water contents, the denser [NTf_2_]^−^–rich phase becomes progressively depleted of both water and acetate. The minimum water concentration in the [NTf_2_]^−^–rich phase depends on the cation's water affinity. This threshold occurs at *x*(H_2_O) = 0.20 for [C_4_C_1_Im]^+^ and at *x*(H_2_O) = 0.29 for ([(C_3_O)C_1_Im]^+^. As previously reported,^[^ ^9^ ^]^ the inclusion of an oxygen atom in the cation's side chain significantly enhances the ionic liquid's affinity for water. At *x*(H_2_O) = 0.90, the two ILs are essentially separated, with water being present just as a minor impurity. This impurity can be efficiently removed using well‐established methods, such as high vacuum at mild temperatures. Alternatively, for ILs prone to thermal decomposition, ion exchange resins or anion metathesis can be used.^[^ ^10^ ^]^

Contrary to earlier reports,^[^ ^11^, ^12^ ^]^ the phase behavior of IL mixtures in the presence of water cannot be attributed solely to the hydrophilicity of the anion. Our findings point to a more complex mechanism, where cation‐water and cation‐anion interactions play a crucial role in determining the water concentration at which phase separation occurs.

Cations with more polar side chains, such as the ether‐functionalized imidazolium cations studied here, exhibit increased hydrophilicity compared to those with alkyl chains. However, they do not show a higher demixing point in terms of water content. This behavior is consistent with weaker cation‐anion interactions in ILs containing ether‐functionalized cations, which has been previously attributed to the “coiling effect” of the ether chain.^[^ ^13^ ^]^ In this configuration, the ether side chain preferentially forms intramolecular hydrogen bonds between the ether oxygen and the acidic hydrogen atoms of the imidazolium ring, thereby further weakening cation‐anion interactions.

The diffusion coefficients of each species in the homogeneous and acetate‐rich phase for [C_4_C_1_Im][OAc]_0.5_[NTf_2_]_0.5_ and [(C_3_O)C_1_Im][OAc]_0.5_[NTf_2_]_0.5_ are depicted in Figure 3 a,b, respectively. They reveal that before phase separation, cations and anions diffuse at similar rates, indicating strong interactions between them. This suggests that the addition of water does not significantly disrupt these strong intermolecular interactions. However, after phase separation, the diffusivity changes dramatically. In the acetate‐rich phase, water diffuses the fastest, followed by [OAc]^−^, then the cation and finally [NTf_2_]^−^. In contrast, in the [NTf_2_]^−^–rich phase (Figure S2) the ions exhibit similar diffusivity values, while water diffuses approximately five times faster than the ions.

**Figure 3:**
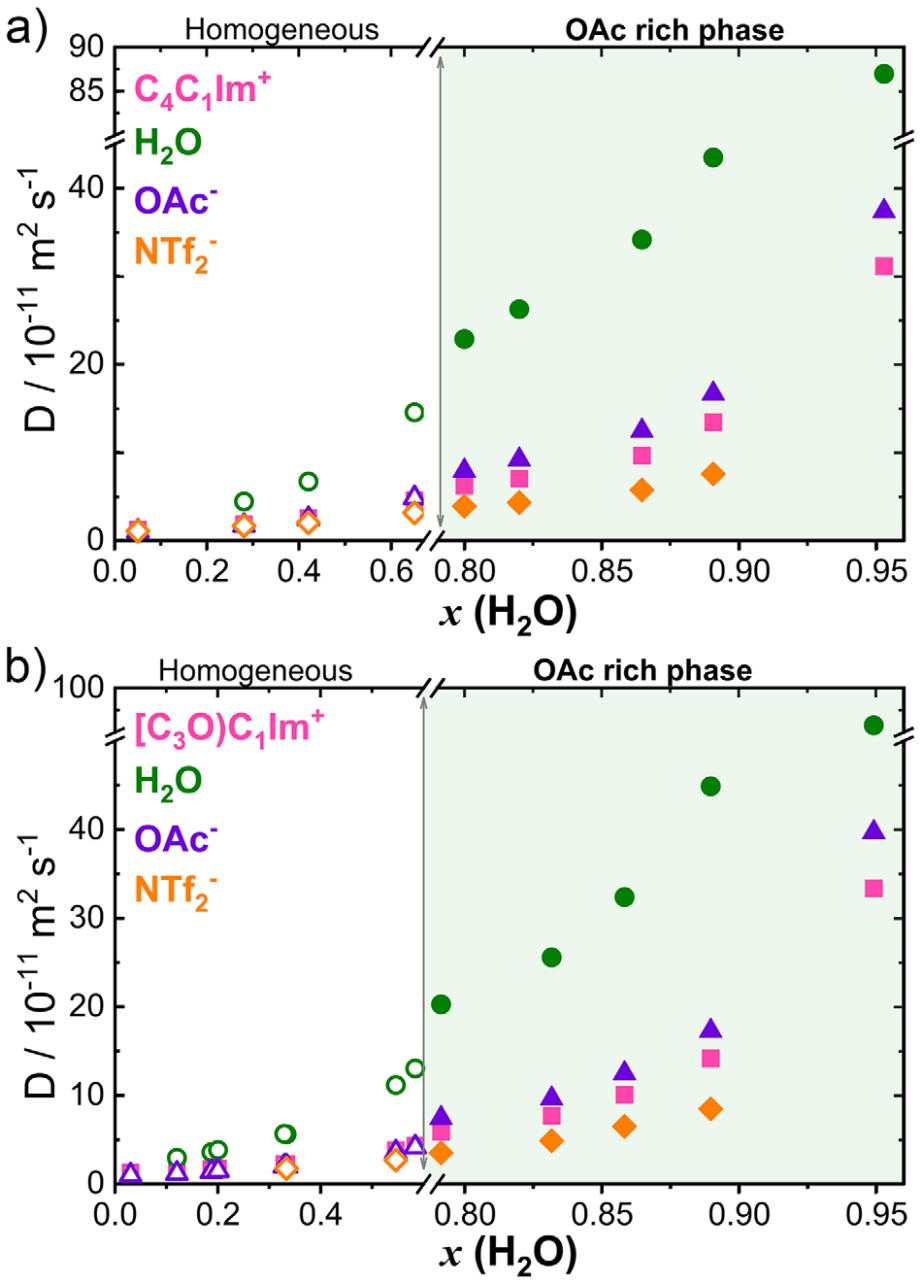
Diffusion coefficient of cation (■), [OAc]^−^ (▲), [NTf_2_]^−^ (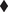) and H_2_O (●) in the homogeneous and acetate‐rich phase (indicated by the green shaded background) for a) [C_4_C_1_Im][OAc]_0.5_[NTf_2_]_0.5_ and b) [(C_3_O)C_1_Im][OAc]_0.5_[NTf_2_]_0.5_.

Interestingly, for both systems, the plot of log (*D*_[OAc]_) versus water mole fraction (Figure S3) reveals two distinct linear regions corresponding to the compositions of the homogeneous and heterogeneous phases. The intersection of these two trends occurs at approximately *x*(H_2_O) = 0.80 (around four water molecules per ion pair), which corresponds to the amount of water required to fully solvate the acetate ion. This finding suggests that the solvation of [OAc]^−^‐IL by water, combined with the low affinity of [NTf_2_]^−^‐IL for water, disrupts the Coulombic interactions that maintain the IL mixture network, thereby driving the phase separation.

The IR spectra, depicted in Figures S4–S9, offer further insights into the interactions between species accompanying phase separation. Adding water causes a noticeable shift to higher wavenumbers for vibrational modes involving hydrogen‐bond interactions, as the acetate ν(CC) and ν(CO) stretching modes, originally observed at ca. 902 and 1386cm^−1^, respectively. Such trend is also observed for the cation stretching CH modes, ν(C(2)‐H) and ν(C(4,5)‐H) at 3010 to 3200 cm^−1^ region, in both homogeneous and acetate‐rich phases, indicating the weakening of cation‐acetate interactions. Conversely, the vibrational modes of [NTf_2_]^−^ at 1348, 1330, 1178, 1132 and 1052 cm^−1^ remain significantly unchanged, indicating that its chemical environment is not affected by the liquid composition. The analysis of the ν_OH_ in both the homogeneous and heterogeneous mixtures, presented in Figure 4 for the most representative compositions, reveals that the bands at ca. 3250 and 3360 cm^−1^ can be defined by the interactions between acetate and water, corresponding to more ordered “ice‐like” and more disordered “liquid‐like” hydrogen‐bond networks, respectively.^[^ ^14^ ^]^ The 3615 cm^−1^ band can be attributed to water molecules shared between [NTf_2_]^−^ and [OAc]^−^ anions, as well as [NTf_2_]^−^–H_2_O interactions.^[^ ^15^ ^]^ In the homogeneous and [NTf_2_]^−^‐rich phases, the higher intensity of the band at 3400 cm^−1^ suggests the disruption of bulk water interactions, whereas in the acetate‐rich phase, the increased intensity of the ice‐like water peak indicates the IL solvation by water. Such structural behavior of water before and after phase separation corroborates the NMR data presented previously. These findings point towards a phase separation predominantly influenced by the salt hydration, as in the Hofmeister series used as the mechanistic basis for elucidating phase separation in aqueous biphasic systems.^[^ ^16^ ^]^

**Figure 4:**
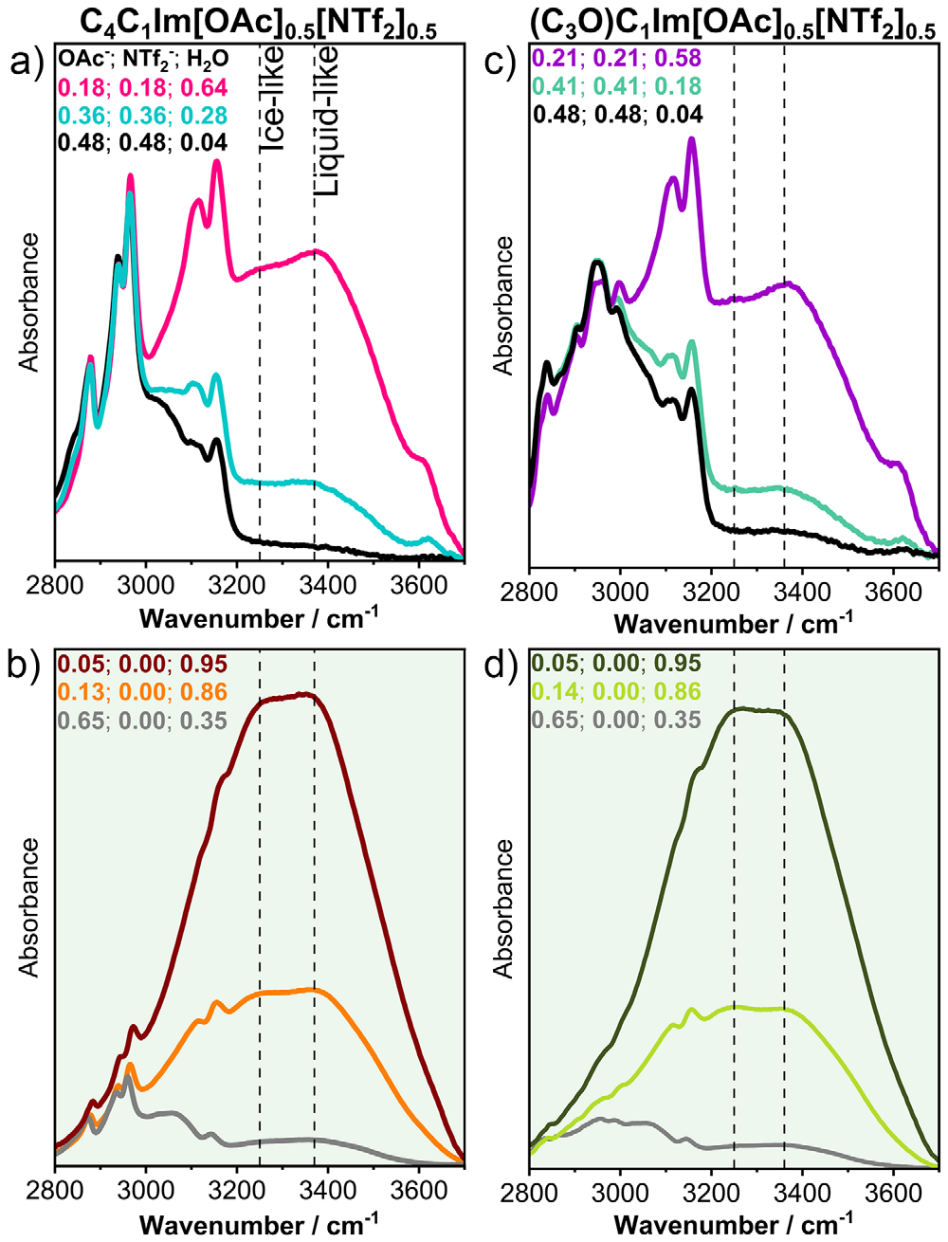
Representative infrared spectra of [C_4_C_1_Im][OAc]_0.5_[NTf_2_]_0.5_ a) b) and [(C_3_O)C_1_Im][OAc]_0.5_[NTf_2_]_0.5_ c), d) at different water mole fractions, highlighting the 2800cm^−1^ to 3700 cm^−1^ spectral region corresponding to the ν(OH) region of water. Panels (a) and (c) are the homogeneous mixture; (b) and (d), with green shaded background, represent the acetate‐rich phase after phase separation, within which the grey curves correspond to [C_4_C_1_Im][OAc] and [(C_3_O)C_1_Im][OAc] mixed with water at *x*(H_2_O) = 0.65 for comparison. Inset labels indicate the sample composition in terms of mole fraction, listed in the order: [OAc]^−^; [NTf_2_]^−^; and H_2_O.

The measured diffusion coefficients and IR spectra reveal the delicate balance of forces and dominant interactions leading to phase separation. First, the demixing point reflects the strength of the interaction between the cation and anion, which is influenced by the water solvation of acetate. Second, the difference in anion hydrophobicity determines the ease of separation of the IL mixture. Finally, the cation's hydrophobicity dictates the purity of each phase.

In summary, the developed method for IL recovery from mixtures demonstrated an efficiency exceeding 99%, providing a sustainable and efficient strategy for IL separation and reuse. We believe this approach establishes a novel pathway for the recovery of ILs from their mixtures–a task traditionally regarded as highly challenging due to the significant risk of sample loss by decomposition or contamination. Indeed, once mixed, such systems have often been described in the literature as forming entirely new ILs, as their recovery was previously considered unreachable. Ongoing studies on other cations and anions, as well as different experimental conditions, aim to provide further insights, particularly into the thermodynamics of phase behavior, which is crucial for optimizing the application of ILs mixtures.

## Conflict of Interests

The authors declare no conflict of interest.

## Supporting information

Supporting Information

## Data Availability

Validation of the NMR quantification data, experimental details, NMR and IR data, as well as numerical data on the composition of each phase for both mixtures are available in Supporting Information.
