# Supplementary material for: Sustainable and Simple Water‐Induced Separation of Ionic Liquid Mixtures
Source: Angew Chem Int Ed Engl. 2025 May 8;64(25):e202503863. doi: 10.1002/anie.202503863 (PMC12171311; doi:10.1002/anie.202503863)
Supplement: Supplementary file 1 — Supporting Information [file ANIE-64-e202503863-s001.pdf]

Supporting Information

Sustainable and Simple Water-Induced Separation of Ionic Liquid Mixtures

B. R. de Moraes,[a,b] A. Pádua,[a] R. A. Ando,[b] and M. Costa Gomes *[a]

[a] *Laboratoire de Chimie de l'ENS Lyon, CNRS and Université de Lyon, 46 allée d'Italie, 69364 Lyon, France. Email: margarida.costa-gomes@ens-lyon.fr*

[b] *Laboratório de Espectroscopia Molecular, Instituto de Química, Universidade de São Paulo, Av. Prof. Lineu Prestes, 748, 05508-000 São Paulo, Brazil. Email: raando@iq.usp.br*

Materials

The ionic liquids (ILs) 1-butyl-3-methylimidazolium acetate (> 98 %) – $[C_4C_1Im][OAc]$ – and 1-butyl-3-methylimidazolium bis(trifluoromethylsulfonyl)imide (99.5 %) – $[C_4C_1Im][NTf_2]$ - were supplied by Iolitec. 1-(2-methoxyethyl)-3-methylimidazolium acetate - $[(C_3O)C_1Im][OAc]$ - was prepared as described by Moraes *et al.*[1] and 1-methoxyethyl-3-methylimidazolium bis(trifluoromethylsulfonyl)imide - $[(C_3O)C_1Im][NTf_2]$ was synthesized[2] and was kindly provided by Dr. Eduards Bakis from the University of Latvia.

The ILs except for $[C_4C_1Im][OAc]$, utilized as received, were dried under vacuum (1×10^{-4} bar) with constant stirring at room temperature for at least 72 h and were kept dried and degassed before the preparation of the IL mixture. The integrity of the liquid was checked by ^{1}H and ^{13}C NMR. The water content of the pure ionic liquids estimated by the Karl Fischer coulometric titration method is listed in Table S1.

Table S1. Water content in ILs.

IL	Water content/ppm	x_{H_2O}
$[C_4C_1Im][OAc]$	7704.7	0.079
$[C_4C_1Im][NTf_2]$	19.8	<0.001
$[(C_3O)C_1Im][OAc]$	5080.2	0.053
$[(C_3O)C_1Im][NTf_2]$	313.2	<0.001

Analysis

Nuclear Magnetic Resonance

The Nuclear Magnetic Resonance (NMR) spectra of ^{1}H and ^{13}C as well as the diffusion coefficient determined by Diffusion Ordered Spectroscopy (DOSY) were measured on a 400 MHz Bruker Avance III spectrometer. The equipment has a Prodigy 5mm 1H-X gradient Z broadband probe at (298.0 ± 0.1) K. The NMR spectra and DOSY for ^{19}F nucleus were collected on a 300 MHz Bruker Avance spectrometer equipped with a broadband BBFO probe at (297.5 ± 0.1) K .

Infrared

The infrared (IR) spectra were collected using a PerkinElmer Spectrum 65 FT-IR spectrometer using the attenuated total reflection (ATR) mode featuring a Germanium crystal and a KBr beam splitter. The measurements were performed by placing a drop of the IL on the ATR crystal. Data were collected in a range of 750–4000 cm^{-1} with a resolution of 4 cm^{-1} and 16 scans.

Preparation of IL mixture

ILs mixtures were prepared in glass vials using a Mettler Toledo New Classic MS balance with an accuracy of ±0.01 mg under an argon atmosphere. The mixtures were stirred at 313 K for 12 h to ensure thorough mixing and then stored in a glove box. All samples formed a homogeneous mixture. The water content of the IL mixtures was determined by Karl-Fischer coulometric titration. The mixtures' water content and calculated molar mass are presented in Table S2.

Table S2. Water content in the ILs' mixture.

IL mixture	Molar mass/g mol^{-1}	Water content/ppm	$x(H_2O)$
$[C_4C_1Im][OAc]_{0.5}[NTf_2]_{0.5}$	309.2	2618.3	0.043
$[(C_3O)C_1Im][OAc]_{0.5}[NTf_2]_{0.5}$	310.3	2253.1	0.037

The water utilized to prepare the ILs' mixture was ultra-pure 15.0 $M\Omega\,cm^{-1}$ and treated with a Purelab Optica water purification device. The mixtures with water were prepared outside the glovebox, and the composition of each prepared mixture (IL mix + H_2O) is detailed in Table S3. The calculated mole fractions shown in Table S3 account for the water content in the IL mixture presented in Table S2.The mixtures with water were vigorously stirred for at least 4 h at 298 K.

Table S3. Sample composition of the prepared IL mixtures.

$[C_4C_1Im][OAc]_{0.5}[NTf_2]_{0.5}$			
$x(H_2O)$ prepared	$x(H_2O)$ by NMR	IL mixture / g	H_2O / g
0.27	0.28	2.19397	0.04206
0.40	0.41	1.86817	0.07274
0.64	0.65	1.14610	0.11443
0.68	heterogeneous	1.14741	0.14079
0.71	heterogeneous	1.14818	0.15830
0.75	heterogeneous	1.14477	0.20527
0.80	heterogeneous	1.15716	0.26672
0.90	heterogeneous	1.35462	0.73565
$[(C_3O)C_1Im][OAc]_{0.5}[NTf_2]_{0.5}$			
$x(H_2O)$ prepared	$x(H_2O)$ by NMR	IL mixture / g	H_2O / g
0.12	0.12	1.14452	0.00992
0.19	0.19	1.07104	0.01231
0.32	0.33	0.64820	0.01586
0.33	0.34	1.00421	0.02566
0.52	0.54	0.35287	0.01310
0.57	0.58	0.46987	0.03228
0.65	heterogeneous	1.51107	0.16523
0.71	heterogeneous	1.42474	0.20098
0.75	heterogeneous	1.45615	0.25382
0.80	heterogeneous	1.40804	0.32763
0.90	heterogeneous	1.31604	0.64800

In the case of samples exhibiting phase separation, the system was initially mixed and subsequently permitted to reach equilibrium for a minimum of 12 h at 298 K. Following equilibration, each phase was meticulously extracted using a needle and syringe for individual analysis by ^{1}H and ^{13}C-NMR, infrared spectroscopy, and Karl Fischer titration. Any additional purification steps were then carried out. The composition of each phase was determined by means of ^{1}H-NMR, with the areas of the peaks corresponding to acetate, water and the cation being integrated and normalized based on the number of hydrogen atoms contributing to each peak. As the anion $[NTf_2]^-$ does not exhibit any ^{1}H-NMR peaks, its quantity was indirectly calculated by subtracting the number of acetate molecules from the cation to ensure the charge neutrality of the system. This normalization facilitated the determination of the relative proportions of each species in the medium. The resulting values were subsequently converted to mole fraction, which directly gives the separation efficiency of the method.

Validation of water content determined by ^{1}H-NMR

To validate NMR as a quantitative method for determining water content in the IL mixture, the values obtained for both the homogeneous phase and the $[NTf_2]^-$-rich phase of $[C_4C_1Im][OAc]_{0.5}[NTf_2]_{0.5}$ were compared with those determined by coulometric Karl Fischer (KF) titration, Table S4. For the homogeneous phase, the mole fraction of water was calculated using the molar mass of the mixture while in the heterogeneous phase was

calculated using the molar mass of $[C_4C_1Im][NTf_2]$. A regression line was fitted to the experimental data using the least squares method with a 95 % confidence interval, yielding an intercept of 0.0605 ± 0.0056 and a slope of 0.9177 ± 0.0162 (Figure S1).

Table S4. Water content in the $[C_4C_1Im][OAc]_{0.5}[NTf_2]_{0.5}$ mixture determined by Karl Fischer and ^{1}H-NMR.

$x(H_2O)$ prepared	$x(H_2O)$ by KF	$x(H_2O)$ by ^{1}H-NMR
0.64	0.64	0.65
0.68	0.32	0.35
0.71	0.23	0.27
0.75	0.15	0.22
0.80	0.15	0.20

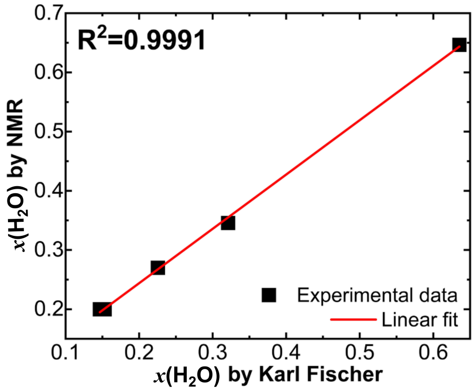

Figure S1. Validation of ^{1}H-NMR as a quantitative method.

Diffusion coefficient by NMR

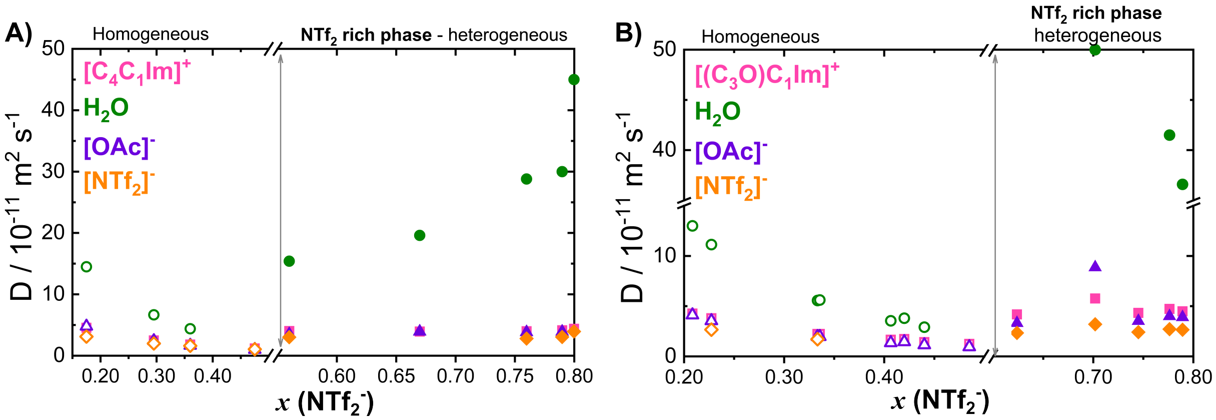

Figure S2. Diffusion coefficient of cation (■), $[OAc]^-$ (▲), $[NTf_2]^-$ (♦) and H_2O (●) in homogeneous and $[NTf_2]^-$-rich phase for **(A)** $[C_4C_1Im][OAc]_{0.5}[NTf_2]_{0.5}$ and **(B)** $[(C_3O)C_1Im][OAc]_{0.5}[NTf_2]_{0.5}$.

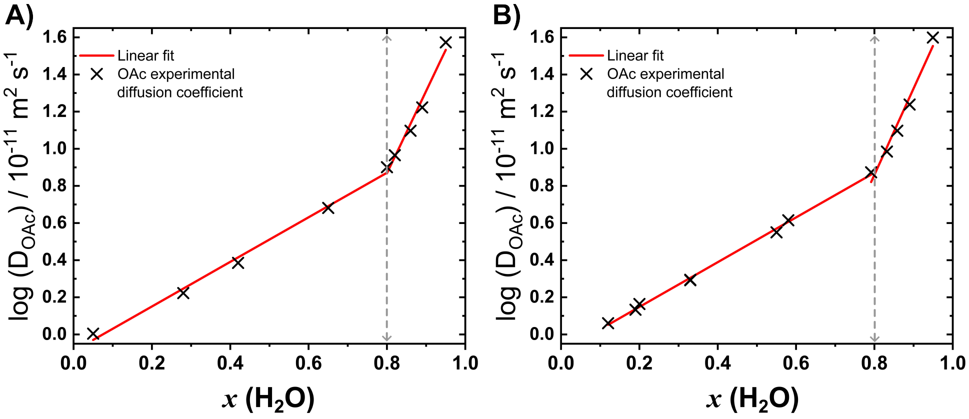

Figure S3. The logarithm of the acetate self-diffusion coefficient in **(A)** $[C_4C_1Im][OAc]_{0.5}[NTf_2]_{0.5}$ and **(B)** $[(C_3O)C_1Im][OAc]_{0.5}[NTf_2]_{0.5}$ at different water mole fractions. The dashed arrow indicates the separation between homogeneous and heterogeneous phases, and the solid red lines represent linear fits for both regimes.

Infrared measurements

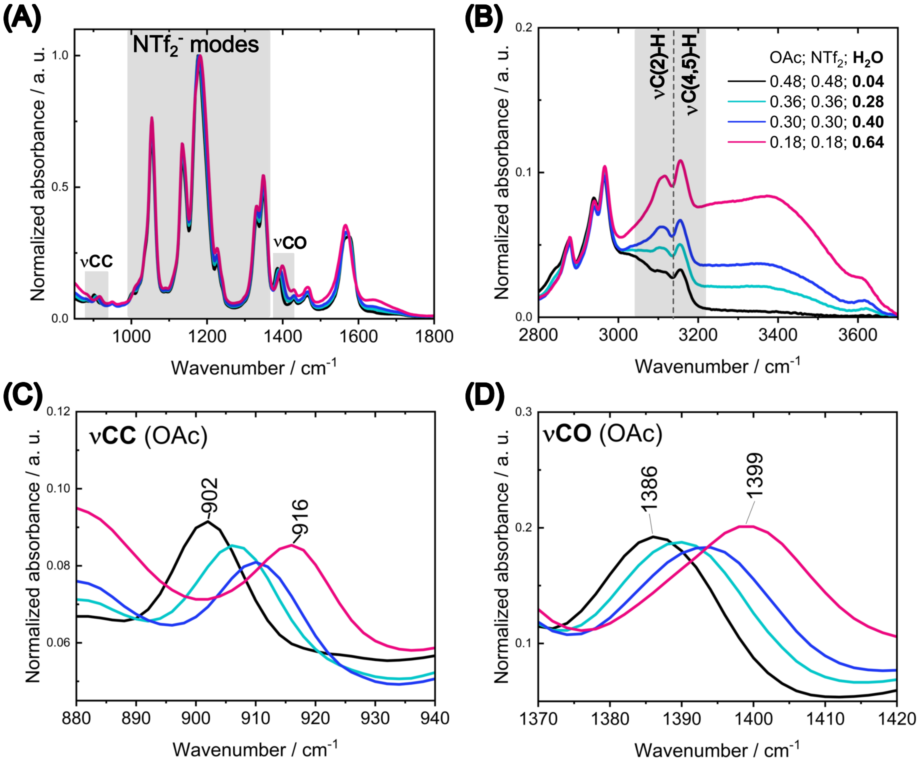

Figure S4. Infrared spectra of $[C_4C_1Im][OAc]_{0.5}[NTf_2]_{0.5}$ at different water mole fractions in the homogeneous phase, presented for the spectral ranges of **(A)** 750–1800 cm^{-1} and **(B)** 2800–3700 cm^{-1}. The spectra are normalized to the most intense band. Panels **(C)** and **(D)** provide magnified views to enhance the visualization of the $\nu(CC)$ and $\nu(CO)$ vibrational modes.

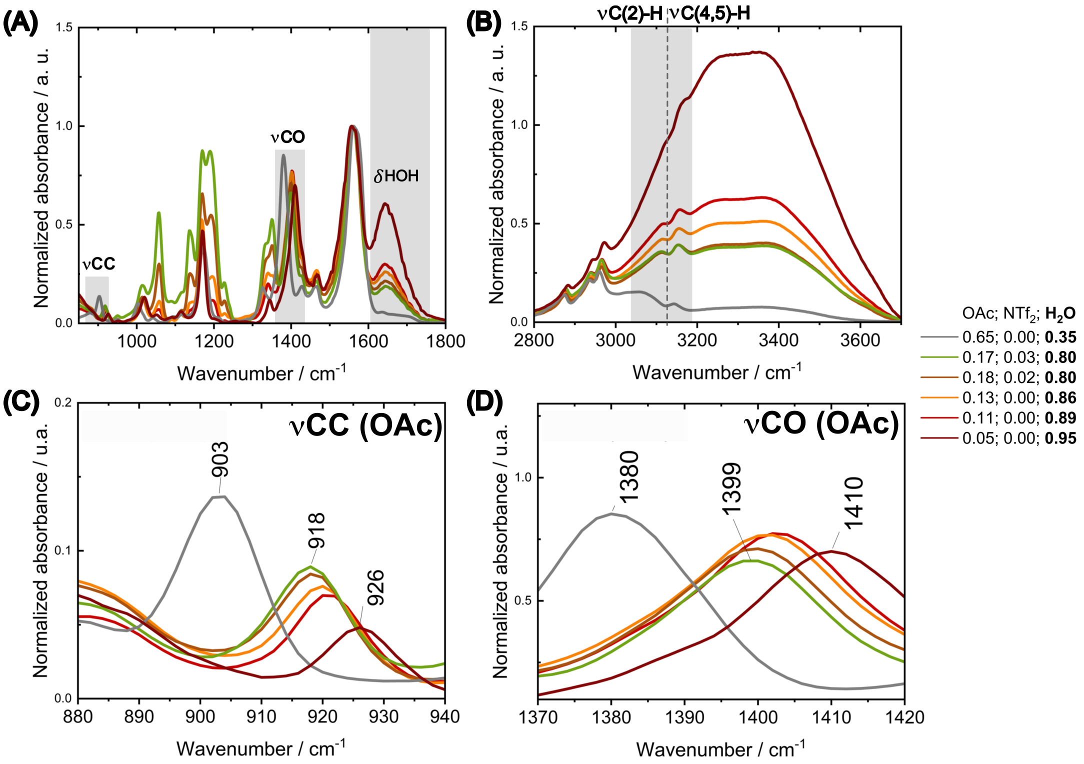

Figure S5. Infrared spectra of $[C_4C_1Im][OAc]_{0.5}[NTf_2]_{0.5}$ at different water mole fractions in the OAc^--rich phase, presented for the spectral ranges of **(A)** 750–1800 cm^{-1} and **(B)** 2800–3700 cm^{-1}. The spectra are normalized to the most intense band. Panels **(C)** and **(D)** provide magnified views to enhance the visualization of the $\nu(\mathrm{CC})$ and $\nu(\mathrm{CO})$ vibrational modes. In all spectra in gray is the homogeneous mixture of $[C_4C_1Im][OAc]$ and water, $x(\mathrm{H_2O}) = 0.35$, for comparison purposes.

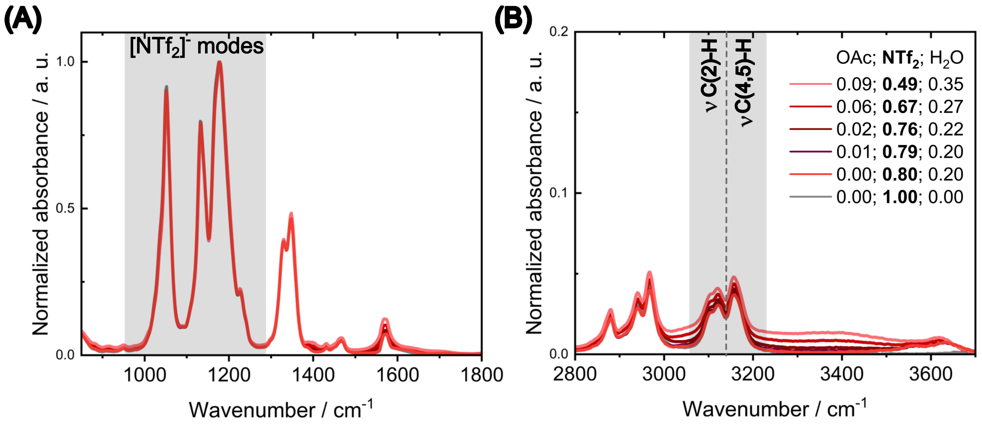

Figure S6. Infrared spectra of $[C_4C_1Im][OAc]_{0.5}[NTf_2]_{0.5}$ at different water mole fractions in the $[NTf_2]^-$-rich phase, presented for the spectral ranges of **(A)** 750–1800 cm^{-1} and **(B)** 2800–3700 cm^{-1}. The spectra are normalized to the most intense band. In all spectra in gray is the neat $[C_4C_1Im][NTf_2]$ for comparison purposes.

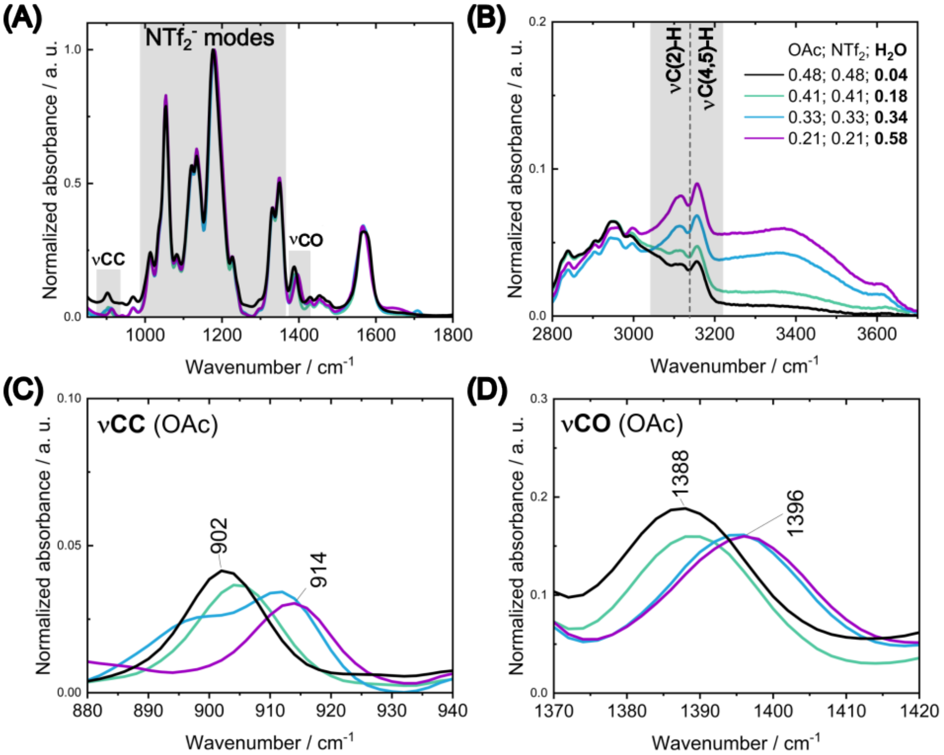

Figure S7. Infrared spectra of $[(C_3O)C_1Im][OAc]_{0.5}[NTf_2]_{0.5}$ at different water mole fractions in the homogeneous phase, presented for the spectral ranges of **(A)** 750–1800 cm^{-1} and **(B)** 2800–3700 cm^{-1} The spectra are normalized to the most intense band. Panels **(C)** and **(D)** provide magnified views to enhance the visualization of the $\nu(\mathrm{CC})$ and $\nu(\mathrm{CO})$ vibrational modes.

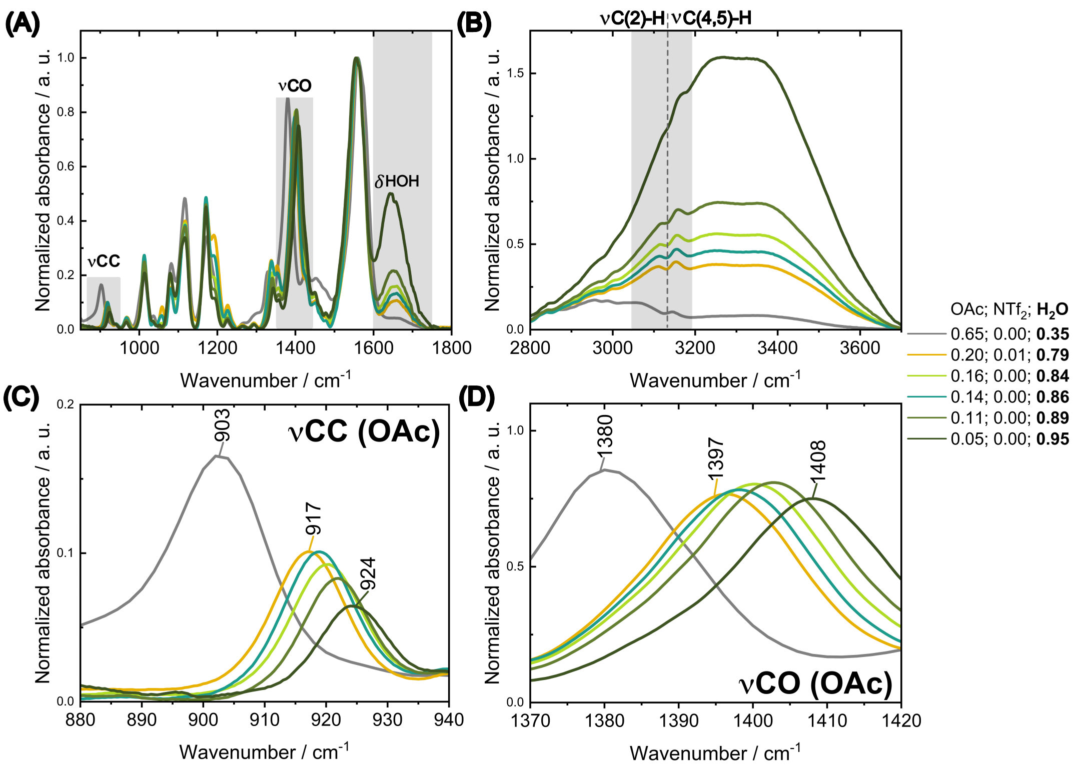

Figure S8. Infrared spectra of $[(C_3O)C_1Im][OAc]_{0.5}[NTf_2]_{0.5}$ at different water mole fractions in the $[OAc]^-$-rich phase, presented for the spectral ranges of **(A)** 750–1800 cm^{-1} and **(B)** 2800–3700 cm^{-1}. The spectra are normalized to the most intense band. Panels **(C)** and **(D)** provide magnified views to enhance the visualization of the $\nu(CC)$ and $\nu(CO)$ vibrational modes. In all spectra in gray is the homogeneous mixture of $[(C_3O)C_1Im][OAc]$ and water, $x(H_2O) = 0.35$, for comparison purposes.

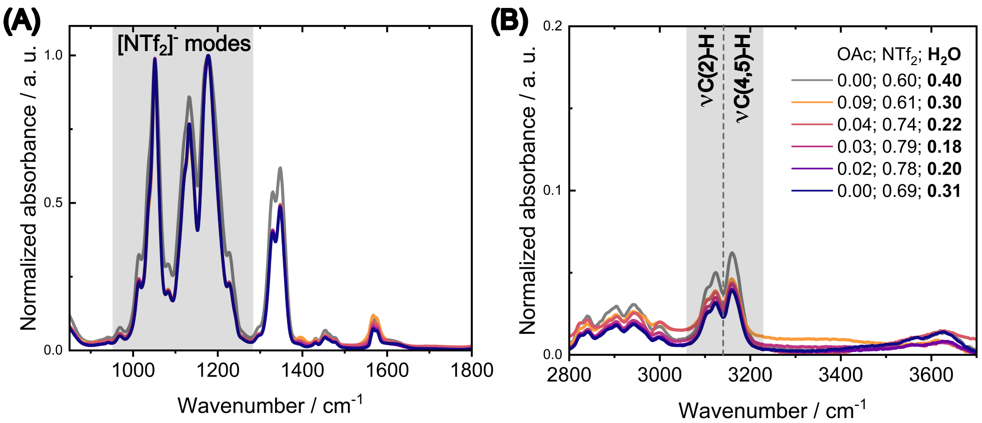

Figure S9. Infrared spectra of $[(C_3O)C_1Im][OAc]_{0.5}[NTf_2]_{0.5}$ at different water mole fractions in the $[NTf_2]^-$- rich phase, presented for the spectral ranges of **(A)** 750–1800 cm^{-1} and **(B)** 2800–3700 cm^{-1}. The spectra are normalized to the most intense band. In all spectra in gray is the $[(C_3O)C_1Im][NTf_2]$ saturated with water, $x(H_2O) = 0.40$, for comparison purposes.

Notes and references

1 B. R. de Moraes, V. H. Paschoal, N. Keppeler, O. A. El Seoud, R. A. Ando, *J. Phys. Chem. B* **2024**, *128*, 4759.

2 E. Bakis, A. van den Bruinhorst, L. Pison, I. Palazzo, T. Chang, M. Kjellberg, C. C. Weber, M. C. Gomes, T. Welton, *Phys. Chem. Chem. Phys.* **2021**, *23*, 4624.
